# Comparative Study on the Efficiency of Using Pulsed and Direct Current Electrochemotherapy in Treating Ehrlich Tumor

**Published:** 2012-03

**Authors:** Mamdouh M. Shawki, Mohammed A. Elblbesy, Thanaa E. Shalaby, Metwali A. Qotb, Youssef S. Youssef

**Affiliations:** *Medical Biophysics Department, Medical Research Institute, Alexandria University, Egypt*

**Keywords:** direct current, low electric field, electrochemotherapy, bleomycin

## Abstract

The Electrochemotherapy (ECT) is an effective treatment of cutaneous and subcutaneous solid tumors. Electric field is applied to tumor nodules to enhance the delivery/distribution of a non-permeable or poorly permeable chemotherapeutic agent into the tumor cells thereby increasing local concentration of anticancer drugs. The aim of this study was to evaluate the effectiveness of using two types of electric fields in ECT, pulsed sine waves and direct current (DC) application in addition to intra-tumoral injection of bleomycin (BLM), a cytotoxic drug for treating Ehrlich tumor. The electric fields were delivered through six stainless steel needle electrodes inserted into the tumor. Tumor volume, tumor mass, percentage of fragmented DNA in the tumor tissue, relative spleen mass/total body mass, mortality rate, histological and ultrastructural examinations were investigated in each group. There were 40% complete response (CR) and 60% partial response (PR) in the group treated with DC as the electric field source of ECT, while 0% (CR) and only 25% (PR) were found in the group treated with pulsed sine wave ECT. We concluded that the utilization of low dose DC for ECT gives better results than the low voltage pulsed sine waves in treating Ehrlich tumor which may be due to the dual effects of electrochemical reactions evoked by DC application and the anti-cancer activity of BLM.

## INTRODUCTION

Successful treatment of solid tumors by chemotherapy depends on the effective penetration of the therapeutic agent into the target cells in the tumor. To achieve this, the systemically administered agent enters the tumor vasculature and reaches the cancer cells via distribution through the vascular compartment, transport across the microvascular wall, then through the interstitial compartment towards the cancer cells. The final step of this overall process is the penetration of the cytotoxic agents into the cells through the plasma membrane. Polar molecules can poorly penetrate into the cells through the plasma membrane. Combined use of a locally delivered permeabilizing electric pulses has been previously proposed to overcome the permeability barrier of the cell membrane for nonpermeant drugs (1). This treatment, known as electrochemotherapy (ECT), uses short and intense electric pulses that transiently and reversibly permeabilize the cells to chemicals through the process of electroporation. Electroporation is generally defined as formation of transient hydrophilic pores by induction of a high transmembrane potential difference (200 mV) after the exposure of cells to (μs) pulsed electric fields of intensity in the range of 300-3000 V/cm (2). The effectiveness of incorporating agents into cells by electroporation is limited by the short lifetime and the diameter of the electrically induced aqueous pores.

Some authors demonstrated that exposure of cells to trains of low electric fields (LEF) pulsed (sine or square waves) in the range of 20-100 V/cm leads to efficient uptake of macromolecules with molecular weight in the range of 300-2,000,000 Da into cells (3). The uptake of macromolecules does not proceed through electroporation but through an endocytic-like mechanism. Therefore, this electrically induced endocytosis in combination with intratumoral injection of chemotherapeutics can be used to enhance the incorporation of antineoplastic drugs into the cells of the solid tumor. This technique which is a new modality of cancer therapy, is known as LEF-ECT (4, 5).

Low-level direct current (DC) has been used as regional cancer treatment. The currents used have been in the range from μA to tens of mA, delivered continuously, or with interruptions for 24 hours per day, or as a single shot treatment. Treatment time has ranged from five minutes to several hours or even days (6). The usage of DC in ECT (DC-ECT) has been suggested (7). Various of electrode materials, electrode configurations, current levels and therapeutic schedules have been employed to date. In the majority of the previous works, one of the metal needle electrodes have been paced into the tumor while the other needle(s) or larger plate electrode placed away from the tumor. Alternatively, in some studies both were placed into the tumor.

In previous work, we optimized the electrode configuration required for this experiment (8). In this experiment we compared between the effects of LEF-ECT and DC-ECT using bleomycin (BLM) as the cytotoxic drug in treating Ehrlich tumor.

## MATERIALS AND METHODS

Experimental Animals: Albino mice weighting 20-25 g of 8-10 weeks of age were used in the experiment. The mice were kept in ordinary light conditions (12 hours light/ 12 hours dark) in constant temperature at 23°C. Induction of Ehrlich tumor in all the mice was developed by injection of 2 × 10^6^ tumor ascites in 100 μl of phosphate buffer saline subcutaneously in the upper right limb of each mouse (9).

Anticancer drug and method of injection: BLM, 8 units of BLM / kg of mouse body weight, was injected intratumorally (10). BLM (Kayaku Company, Japan) is a positively charged and therefore it will be attracted by the negative electrode (11).

**Figure 1:**
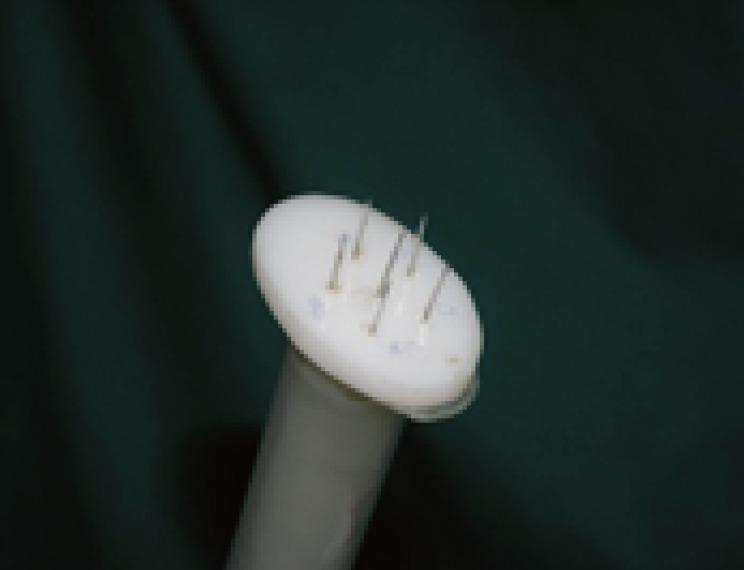
Six electrodes configuration used in this study.

Six electrodes arrangement (Fig. 1) were manufactured using stainless steel needles of 0.3 mm diameter and 6 mm length. The electrodes were arranged in a circle with the cathode at the center and the other five electrodes on the circumference worked as anodes. The distance between each electrode and the other was 6 mm. The electric circuits: Two electrical circuits were constructed locally; one to produce continuous DC with a regulating facility (rheostat). The other circuit was constructed to produce LEF pulsed sine waves; 50 Hz, with pulse duration of 10 ms, the field strength was controlled by using a rheostat. The electric fields were delivered into the tumor through the electrodes where the cathode was in the center of the tumor.

### Application of ECT protocol

When tumors reach 200-250 mm^3^ in volume, 180 Ehrlich tumor-bearing mice were randomly classified into six groups; 30 mice each, mice were anaesthetized (systemic anaesthetized using intramuscular mixture of ketamine and xylazine, 1 ml/10g body weight, Sigma company) for all exposure conditions. The groups were classified as follows:

G0: injected with 0.2 ml saline only and served as control.
GI: injected with 0.2 ml BLM intratumorally and after 2 minutes, the tumor was exposed to LEF pulsed sine waves 3.6 V/cm for 12 minutes.
GII: exposed to LEF pulsed sine waves 3.6 V/cm for 12 minutes.
GIII: injected with 0.2 ml BLM intratumorally and after 2 minutes, the tumor was exposed to continuous DC electric field 2.5 V/cm for 30 minutes.
GIV: exposed to continuous DC electric field 2.5 V/cm for 30 minutes.
GV: injected with 0.2 ml BLM intratumorally only.

After the application of the treatment protocol, each group was equally divided into 5 subgroups, weighted and sacrificed at time intervals of; the same day of treatment, 2, 9, 18 and 120 days after the treatment

After the exposure of each group to the corresponding exposure protocol, each group was subdivided into five subgroups as follows:

1. Five mice anaesthetized and sacrificed at the same day of exposure.
2. Five mice anaesthetized and sacrificed after two days of exposure.
3. Five mice anaesthetized and sacrificed after nine days of exposure.
4. Five mice anaesthetized and sacrificed after eighteen days of exposure.
5. Ten mice kept without sacrificing for survival calculations up to 120 days after exposure.

### Measuring of tumor volume and its growth

Tumor volume is an important indicator of its growth dynamics. The measurement takes place by vernier caliper and then applying the following equation (12):

(a) $$V\left(\mathrm{tumor}\right)=\left(\pi^{*}a^{*}b^{2}\right)/6$$

Where a, b are orthogonal diameters (length and width respectively).

### Determination of tumor mass

The tumor was separated from the fatty tissue around it then washed three times using normal saline then weighted by digital balance (error 0.1 mg, Sartorius AG company, Germany).

### Relative spleen mass to total body weight r (m)

It’s a primary method for notification of induction of high immune response against certain body reaction. The spleen was separated from the surrounding fat tissues and washed by saline then with distilled water, left on a filter paper for 15 minutes, weighted accurately, and then the relative spleen mass was calculated as follows:

(b) Spleen r m=mspleen/Wmouse

### DNA fragmentation test

DNA fragmentation test was done in order to determine the percent of apoptotic cells/0.1 g tumor tissue the standard method of Diphenylamine solution was used (13).

### Histological studies

Histological studies was performed by using the standard method of Haematoxylin and Eosin (H&E) stain (14), while the image analysis was done by the soft imaging system (Digital imagining, Olympus Company, USA).

### Ultrastructural studies

The standard specimen preparation method for transmission electron microscope was used (15) and then the samples were examined by using an electron microscope (TEM, 100CX, JEOL, USA) (15).

### Survival time calculations

Mortality rate in each group was given in percentage and calculated by using the following equation:

(c) $$\%\mathrm{mortality}=N\left(number of dead mice\right)/N{\left(total number of animals in the group\right)}^{*}100$$

### Statistical analysis

The data analysis was performed using the SPSS-10 package (release 3, SPSS Inc., Chicago III) running on MCROVAX 3500. ANOVA test was used to compare between the means of the different parameters of the samples of the six studied groups. Paired t- test was used to compare the significant difference within the subgroups of the same group. A difference was considered significant at probability (*p*<0.05).

## RESULTS

**Figure 2:**
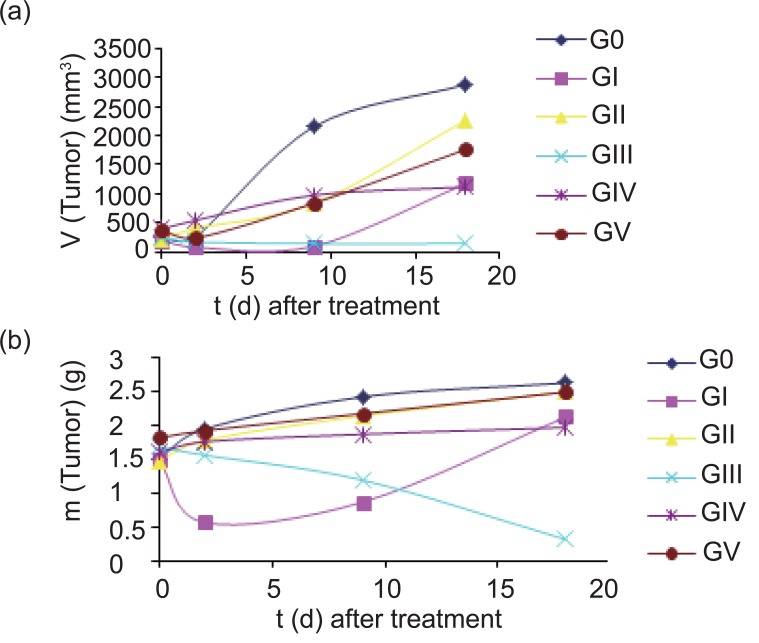
Quantitative measurements of the tumors against time (days) after treatment (a) tumor volume (b) tumor mass.

**Figure 3:**
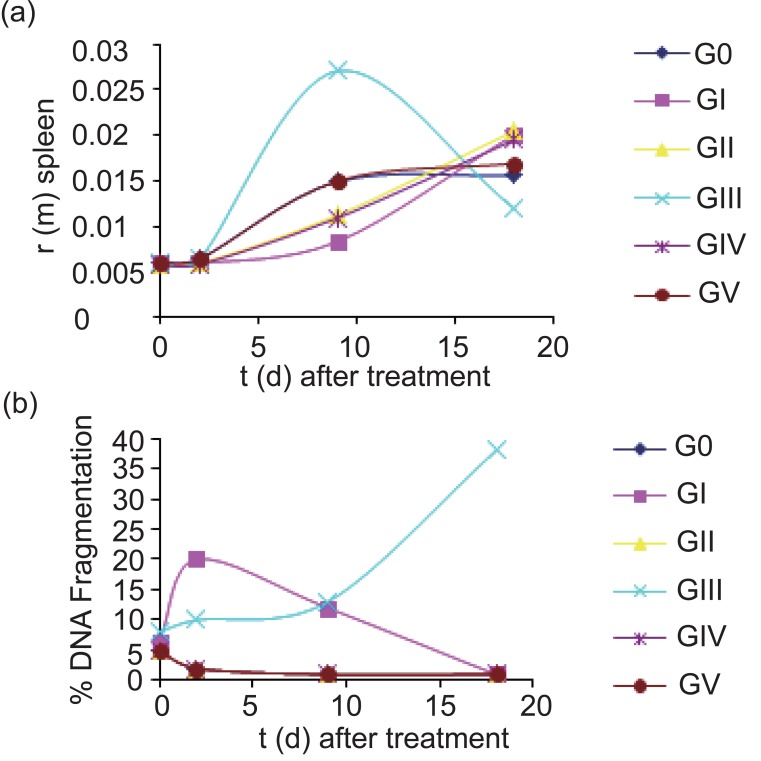
(a) Relative spleen mass/total body weight r (m) against time (days) after treatment (b) percent of DNA fragmentation/0.1 g tumor mass against time (days) after treatment.

We first examined the tumor growth dynamics through the measurements of tumors volume and mass in each group (Fig. 2a-b), the data show a significant decrease in both tumor volume and mass in GIII with time after treatment compared to control group. The induction of high immune response was studied by measuring the relative spleen mass/total body weight r (m) for each group (Fig. 3a), the results showed that the spleen r (m) was significant increase in GIII after 9 days of treatment then significantly decreased again with days after treatment. The tumor apoptosis was indicated by determination the percent of DNA fragmentation/0.1 g tumor mass (Fig. 3b), there was a significant increase in the fragmented DNA percent with days after treatment in GIII, also a significant increase in the percent of fragmented DNA was found in GI after 2 days of treatment but this percent is significantly decreased again with days after treatment.

**Figure 4:**
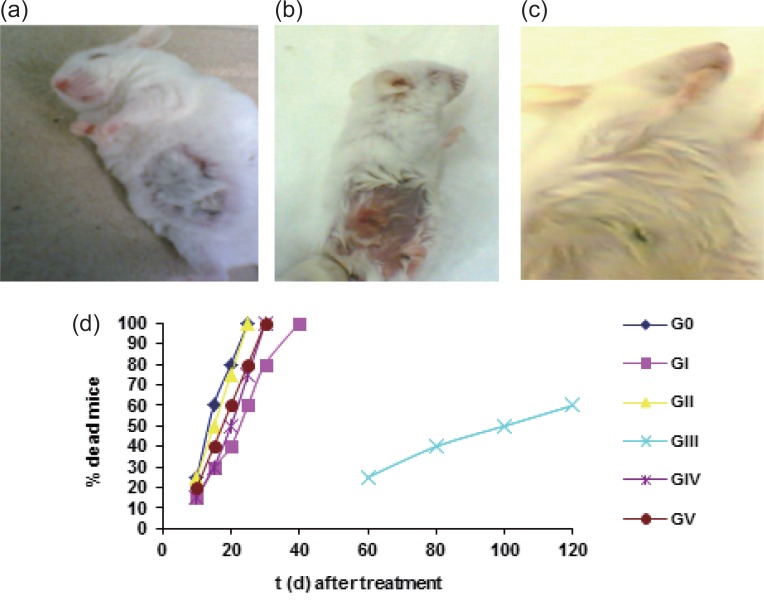
“a-c” Tumor photos after 18 days of treatment (a) big volume for G_0_ (b) decrease in tumor volume for GI (c) no tumor mass can be detected for GIII. (d) The percent of dead mice against time (days) after treatment.

After 18 days from the application of the treatment protocol, the tumor region for 6 random mice from each group was photographed with digital camera. The photos showed big tumor region in the control group (Fig. 4a) (V= 2877 ± 608 mm^3^). A decrease in the tumor region in GI and GIV compared with control (Fig. 4b) (V= 1177 ± 583 and 1120 ± 279 mm^3^ respectively), while very small tumor region could be detected in the photos in GIII (Fig. 4c) (V= 151 ± 80 mm^3^). The percent of mice death (Fig. 4d) indicated that 40% of GIII mice were alive after 120 days from the treatment.

**Figure 5:**
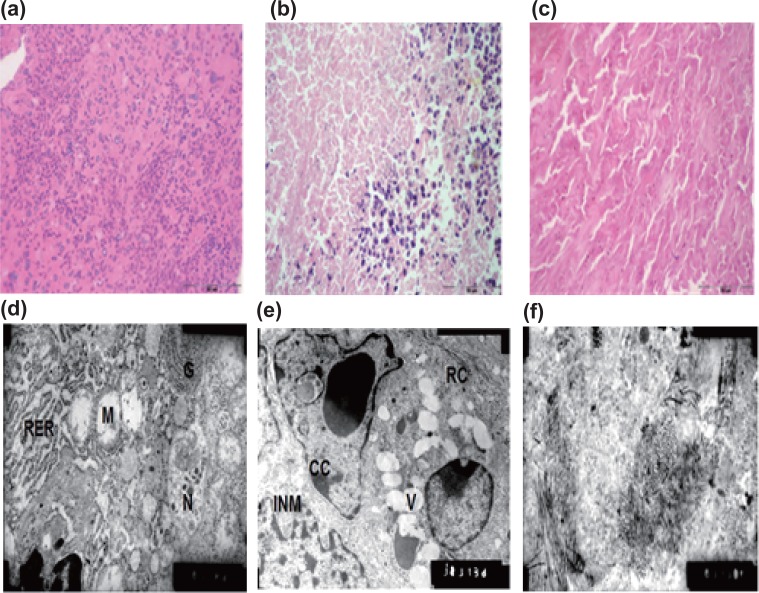
**“a-c”**: Light micrographs (H&E, 400X) after 18 days of treatment: (a) active malignant nuclei for G_0_ tumor (b) some active nuclei and some necrotic area for GI tumor (c) complete necrotic area for GIII tumor. “d-f”:electron micrographs (10000X) after 18 days of treatment (d) for GI tumor: irregular nuclear membrane (INM), clumped chromatin (CC), ruptured cytoplasm (R C), degenerative organelles and many vacuoles (V) were found (e) for GIV tumor: some dead cells appeared with swollen cytoplasm, the mitochondria (M) were large, swollen and with degenerated cristae, diffused rough endoplasmic reticulum (RER), fragmented nuclei (N) and prominent Golgi apparatus (G) (f) for GIII tumor: complete necrotic area.

Histological examinations were performed to determine the degree of tumor prognosis in each group. The control group showed high activity of tumor cells with multinuclear active malignant nuclei (Fig. 5a). The results of GI showed apoptosis in some tumor cells while active malignant nuclei were also appeared; similar results found in GIV (Fig. 5b), while there was massive necrosis with complete area of cell death in GIII tumors (Fig. 5c).

The ultrastructural study indicated that there were some pyknotic dead cells in GI (Fig. 5d); similar results were obtained in GIV (Fig. 5e), while there was complete tumor death in GIII (Fig. 5f).

## DISCUSSION

In the present work, we examined two different electric fields for ECT method; LEF- ECT which was examined by other authors before (3-5), and DC-ECT which is a new technique. The used cytotoxic drug was BLM, and the tumor model was Ehrlich tumor. In addition we examined the effect of each one of the electrical fields. DC-ECT results are significantly better efficiency than LEF-ECT. The cause of the difference may be due to the electrochemical reactions evoked by DC application. Those reactions might have induced a disturbance in the cell membrane and apoptosis, both of which would lead to cell death. So the combination of DC with BLM increases apoptosis which in turn activates the immune response with the increase in spleen r(m) to perform necrosis accompanied with the increase in the fragmented DNA percent (Fig. 3a-b).

There is a possible suggested mechanism for pulsed LEF-ECT that the induced endocytosis based on electrophoretic lateral mobility of charged proteins and lipids in the plasma membrane which induces electrophoretic lateral mobility of charged proteins and lipids in the plasma membrane of adherent cells (16). A similar phenomenon takes place when large membrane vesicles in suspension are exposed to pulsed LEF. That electrophoretic mobility brings an unselective increase in protein-protein association and the formation of segregated domains of lipids and proteins which leads to the stimulation of endocytic pathways. A complementary explanation may be based on the claim that imbalance of charge distribution in the two opposite lipid leaflets of the cell membrane will result in destabilization of surface energy, leading to enhanced membrane undulation resulting in enhanced membrane vesiculation (17).

DC treatment of the tumors was previously examined and showed a significant success. Its mechanism depends on the change in the tumor pH and production of free radicals which lead to wide destruction of some parts of the tumor membrane (18). According to Harguindey (19), tumor hyperacidification might activate cytolytic mechanisms via increased activity of lysosomes, resulting in destruction of tumor tissues. Low pH also inhibits glycolysis and protein synthesis, upon which malignant tissues are dependent. The hyperacidification and the decrease in tumor pH can explain the prognosis achieved in GIV (treatment with DC only) (Fig. 5e). The produced free radicals act to oxidize the lipid bilayer of the cell membrane, and in turn the cellular membranes of the tumor become disturbed and temporary loss its proper selectivity functions, and the cytotoxic drug can overcome the membrane barrier, and this is the main idea of DC-ECT.

The transport of bleomycin across the nonpermeabilized plasma membrane is achieved by carrier proteins that internalize it by the endocytotic pathway, but that transport is limited by the low number of carrier proteins. Using LEF-ECT or DC-ECT to increase membrane permeability provides BLM with direct access to cytosol and transport to DNA. The cytotoxicity of BLM is thereby increased by a multiple of several thousand (20).

DC-ECT results are significantly better than LEF-ECT and that may be because electrochemical reactions caused by DC help in both tumor death (apoptosis) and disturbance of tumor cell membranes.

The results of GI indicated that firstly, there was an increase in the fragmented DNA percent then the percent decreased with time after treatment (Fig. 3b), also the histological and ultrastructural pictures (Figs. 5b and 5d) emphasized that the tumor was not completely destroyed. These results may be explained as LEF-ECT depends on the BLM endocytosis only, in case that BLM cannot enter to any malignant cell (due to the incomplete coverage of the tumor with the electric field or the shortage of the exposure period), this cell may develop even low numbers of new malignant cells and the tumor start to grow again. This problem can be overcome in the future work by using multiple doses of the exposure at different time intervals to give better chance for BLM endocytosis.

The electrode material is also an important factor. A positive potential on metal electrodes leads to corrosion of the metal with the release of metal ions from the electrode and possible resultant necrosis due to metal toxicity. At the anode the stainless steel electrode is corroded with the ferrous ions going into solution. Also ferrous is the cofactor of BLM and this is the reason for using stainless steel electrodes in this paper.
